# Lead Removal Without Extraction Tools: A Single-Center Experience

**DOI:** 10.21470/1678-9741-2018-0275

**Published:** 2019

**Authors:** Neiberg de Alcantara Lima, Gisele Schinaider Cunha, Natalia Soares de Menezes, Evilásio Leobino da Silva Junior, Carol Cavalcante de Vasconcelos Lima, Stela Maria Vitorino Sampaio

**Affiliations:** 1 Western Michigan University, Michigan, United States.; 2 Hospital de Messejana Dr. Carlos Alberto Studart Gomes, Fortaleza, CE, Brazil.; 3 Universidade Federal do Ceará, Fortaleza, CE, Brazil.; 4 Clínica Radiológica Mario de Assis, Fortaleza, CE, Brazil.

**Keywords:** Pacemaker, Artificial, Lasers, Traction, Retrospective Studies

## Abstract

**Introduction:**

Indications for cardiac devices have been increasing as well as the need for lead extractions as a result of infections, failed leads and device recalls. Powered laser sheaths, with a global trend towards the in-creasingly technological tools, meant to improve the procedure’s outcome but have economic implications.

**Objective:**

The aim of this study is to demonstrate the experience of a Bra-zilian center that uses simple manual traction in most lead removals per-formed annually, questioning the real need for expensive and technically challenging new devices.

**Methods:**

This retrospective observational study included 35 patients who had a transvenous lead extraction in the period of a year between January 1998 and October 2014 at Hospital de Messejana Dr. Carlos Alberto Studart Gomes, in Fortaleza, CE, Brazil. Data were collected through a records review. They were evaluated based on age, type of device, dwelling time, indication for removal, technique used and immediate outcomes.

**Results:**

The median dwelling time of the devices was 46.22 months. Infec-tion, lead fracture and device malfunction were the most common indica-tions. Simple traction was the method of choice, used in 88.9% of the pro-cedures. Manual traction presented high success rates, resulting in com-plete removal without complications in 90% of the cases.

**Conclusion:**

This article suggests that lead extraction by simple manual traction can still be performed effectively in countries with economic diffi-culties as a first attempt, leaving auxiliary tools for a second attempt in case of failure or contraindications to the simple manual traction technique.

**Table:** 

Abbreviations, acronyms & symbols	
CIED	= Cardiac implantable electronic devices
CRT	= Cardiac resynchronization therapy
ICD	= Implantable cardioverter defibrillators
LEXICON	= Lead Extraction in the Contemporary Setting study
PM	= Pacemaker

## INTRODUCTION

The indications for cardiac implantable electronic devices (CIED), pacemakers (PM) and implantable cardioverter-defibrillators (ICD) have been increasing due to the aging of the general population and the results of large clinical trials showing benefit for primary prevention of sudden cardiac death^[1,2]^.

In addition, the rising number of comorbidities per patient as well as the use of cardiac resynchronization therapy (CRT) devices, which require more leads to be implanted, contribute to the increasing need for lead extractions as a result of infections, failed leads and device recalls^[3]^.

Even though up to 30,000 lead extraction procedures are performed annually worldwide, no standard approach has been established^[2,4,5]^.

Transvenous lead extraction is the preferred method of treatment of CIED-related complications, as it has improved over time, becoming safer and more effective. It uses methods that vary from simple local traction to the use of powered laser sheaths, with a global trend towards the increasingly technological tools, meant to improve the procedure’s outcomes^[6]^.

The choice to perform an extraction should be weighed carefully, following internationally accepted recommendations and an individualized approach, since these procedures carry the risk of acute morbidity and mortality and may have poor long-term outcomes^[7]^.

Simple manual lead traction was once widely used, but due to the avulsion, laceration or perforation related cases, it became obsolete in many developed countries. In our hospital, a public cardiology center in the Northeast of Brazil, the availability of lead extraction tools is low, then manual lead traction remains an accepted procedure.

## OBJECTIVE

The aim of this study is to demonstrate the experience of a Brazilian Center that uses simple manual traction in most of the lead removals performed annually, questioning the real need for expensive and technically challenging new devices.

## METHODS

This retrospective observational study included 35 consecutive patients who had a transvenous lead extraction in the period of a year between January 1998 and October 2014 at Hospital de Messejana Dr. Carlos Alberto Studart Gomes, Fortaleza, CE, Brazil. Data were collected through a records review.

All the procedures were done by the same expert surgeon in an operative room using a mobile C-Arm image intensifier. Simple manual traction and extraction with Cook extractor were done under local anesthesia plus IV sedation. Open heart surgeries were done under general anesthesia.

Immediately after surgery and 24 hours later, patients had complete blood count, chest radiograph and electrocardiogram done to look for possible complications. Clinical and surgical complications were observed throughout hospitalization and additional exams were requested if needed by the primary cardiology team. Patients had outpatient visits within one week and then after one month. After the initial visits, they were seen every six months in the pacemaker outpatient service. It was defined as a complication related to the procedure: death, infection, hemopericardium, hemothorax, pneumothorax, bleeding requiring blood transfusion and other pulmonary complications.

Simple manual traction technique - After removal of the device and dissection of fibrous tissue around the lead, simple traction of the lead was performed following insertion of a non-locking stylet and retrieval of screws until the separation of the lead from the myocardium and venous system was accomplished.

Non-powered traction tools with Cook kit - After removal of the device and dissection of fibrous tissue around the lead, the locking stylets slide into the lumen of a lead and advance to its tip where they were locked into position, directing the force of traction to the length or at their distal end. Mechanical dilator sheaths were advanced along the lead to disrupt and dilate the fibrotic attachments. If calcified, the single sheath technique often required rotational movement to succeed.

Open heart surgery - a combined transvenous and open surgical extraction approach was undertaken. Median sternotomy was performed, followed by cardiopulmonary bypass and cardiac arrest. Initially, the leads were cut at the superior vena cava level and extracted from the right ventricle and atrium. Then, with the patient out of cardio-pulmonary bypass, the generator and the leads parts from the subclavian to the superior vena cava were withdrawn.

Non-powered traction tools were used depending on the availability of the material in our institution. Open heart surgery was done only when the patients had contraindications to the other procedures, such as concomitant need of surgical repair or large vegetations.

They were evaluated based on age, type of device, dwelling time, indication for removal, technique used and immediate outcomes.

At the end of data collection, 36 procedures were described in 35 patients and their data were tabulated in Microsoft Excel spreadsheets for simple descriptive analysis.

This project was accepted and approved by the local Ethics Committee of the Hospital de Messejana on February 8, 2015. This research did not receive any specific grant from funding agencies in the public, commercial, or not-for-profit sectors.

## RESULTS

The median dwelling time of the devices was 46.22 months, ranging from 0.25 to 180 months. The main indication for removal was infection, accounting for 55.6% of the cases (including patients who had extrusion of the system, with or without associated infective endocarditis, along with those who had isolated infections and one case of septic shock). Lead fracture was the second most common issue (40.06%), followed by device malfunction due to lead noise (3.12%) (Table 1).

**Table 1:** Baseline characteristics.

**Ages (years)**		66.7 (24-95)
**Males**		21 (60%)
**Dwelling time (months)**		46,22 (0.25-180)
**Simple traction (n=32)**		
**Indication for removal**	Extrusion	10/32 (31.25%)
	Extrusion and infectious endocarditis	1/32 (3.12%)
	Infection without extrusion	6/32 (18.75%)
	Lead fracture	13/32 (40.06%)
	Inappropriate shocks due to noise	1/32 (3.12%)
	Septic shock	1/32 (3.12%)
**Device**	ICD	2/32 (6.25%)
	Pacemaker	30/32 (93.75%)
**Outcome/Complication management**	Complete extraction without complication	29/32 (90.62%)
	Complete extraction. Complicated by diaphragmatic palsy without any additional management	1/32 (3.12%)
	Incomplete extraction. Atrial lead not removed. No additional management needed	1/32 (3.12%)
	Incomplete extraction. Complicated by infection followed by an open heart surgery	1/32 (3.12%)
**Open heart surgery (n=2)**		
**Indication for removal**	Extrusion and infectious endocarditis	2/2 (100%)
**Device**	Pacemaker	2/2 (100%)
**Outcome/Complication management**	Complete extraction without complications	1/2 (50%)
	Incomplete extraction without complications	1/2 (50%)
**Non-powered traction tools (n=2)**		
**Indication for removal**	Complete extraction without complications	1/2 (50%)
	Incomplete extraction. No additional management needed	1/2 (50%)
**Device**	ICD	1/2 (50%)
	Pacemaker	1/2 (50%)

ICD=implantable cardioverter defibrillator

**Fig. 1:**
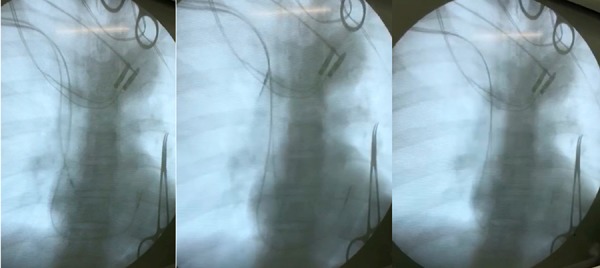
Atrial lead manual traction extraction.

**Fig. 2:**
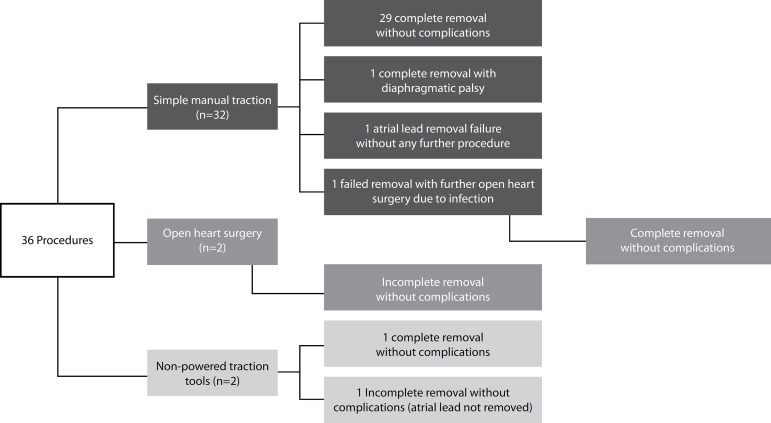
Outcomes of the procedures.

Simple traction was the method of choice, used in 88.9% of the procedures (32). Manual traction (Figure 1) had high success rates, resulting in complete removal without complications in 90,6% of the cases (29 procedures). One patient developed diaphragmatic palsy after the procedure (Figure 2).

Cook extractor was only used twice due to the unavailability of the material in our hospital. One procedure was successful without complications.

Three cases resulted in abandoned leads, two in the manual traction group and one in the Cook Extractor group. One of those who had an initial manual traction failure underwent a surgical procedure 10 months later due to infection.

Open heart surgery was performed in two patients, in both cases due to endocarditis with large vegetations. One patient failed manual traction previously.

## DISCUSSION

CIED infections are the strongest and most common indication for lead removal worldwide, leading to a high risk of death if left untreated^[5]^. They were the most common indication in the population study.

The number of device-related infections have increased disproportionately to the implantation rate, most likely for multifactorial reasons: raised awareness, use of systems that demand more complex procedures (such as ICDs and CRT), as well as the growing number of elderly patients with multiple comorbidities in use of CIED^[5]^.

It is important to point out that an infection of any component of the CIED implies compromise of the entire system. Therefore, once diagnosed, all the components should be removed and antibiotic therapy initiated^[8,9]^.

Lead recalls and malfunctions have increasing the number of extractions, despite improvements in design and performance. Lead failure can represent the breakdown of any of its components, including insulation, conductors, connectors, terminal pins, electrodes, and coils^[2]^.

We observed one case of inappropriate shock due to noise, which usually occurs secondary to device malfunction, as well as 15 cases of lead failures due to fractures.

Factors influencing the lead extraction outcomes are lead implantation duration; lead tip location and properties (presence of defibrillator coils, lead type and insulation material), presence of infection, individual anatomy and physician experience^[4]^.

Fibrosis and adherence are expected complications, for which the challenges and risks associated with lead extraction are mainly related. They preclude lead removal through simple manual traction, as this might cause avulsion, laceration or perforation^[1,7]^.

A large number of extraction tools have been developed to minimize or eliminate complications. The majority of lead extraction procedures were performed from the lead venous entry site but, in cases of failure, femoral or internal jugular approach can be used^[6]^.

Our cardiology center is a public health institution and therefore relies on government resources, which are often scarce. Simple manual traction is the most widely used method since mechanical extractors are costly and not widely available, forcing the surgeons to develop experience in this technique in different scenarios.

Similar conditions were present in the study by Jo et al.^[10]^, who evaluated promptly available tools as alternatives to more expensive methods. Their success rate was 70% using simple manual traction, safer and more effective, specially for the extraction of infected leads and those with a short dwelling time^[10]^.

Paraskevaidis et al.^[11]^ showed success rates above 90% using non-powered traction tools, with minimal complications. In a study that used laser-powered sheaths followed by mechanical tools, patients had complete extraction in more than 95% of the cases, with complications in less than 1%^[12]^. In the PLEXES trial, the efficacy and safety of laser sheaths were tested against conventional lead extraction methods in 301 patients with 465 chronically implanted pacemaker leads and the complete lead removal rate was significantly higher in the laser group and was reported to be up to 94%^[13]^. More recently, the Lead Extraction in the Contemporary Setting (LEXICON) study reported the outcomes of laser-assisted extraction of 2,405 leads in 1,449 consecutive patients and the overall procedural success rate associated with complete lead removal was 96.5% and major complications in 1.4% of the 1449 patients enrolled, with death in 0.28% (deaths caused by vascular tears)^[14]^.

Our success rates with manual traction are satisfactory, even for leads with very long dwell times, despite the well-recognized possible complications of this technique. The few available local resources compel physicians to master techniques that have been traded for innovations in many parts of the world. We thereby demonstrate that classic methods can still be performed safely and effectively.

The complications in our study were a diaphragmatic palsy (minor complication) and endocarditis. When compared to other studies, the rate of complications was acceptable. No death, hemopericardium, pleural effusions or other major complications were observed.

This study has limitations. It was conducted in a single local center and only single surgeon procedures were included. It was observational and did not have a control group.

## CONCLUSION

This article suggests that lead extraction by simple manual traction can still be performed safely and effectively in countries with economic difficulties as a first attempt in selected patients, leaving expensive auxiliary tools for a second attempt in case of failure or contraindications for simple manual traction technique.

Simple manual traction, however, may cause lead damage and it might preclude to use active tools in a second time. Therefore, any traction in the lead must be done with caution and if there is any risk of lead damage the approach must be suspended and the active tools should be used.

**Table:** 

Author's roles & responsibilities	
NAL	Substantial contributions to the conception or design of the work; or the acquisition, analysis, or interpretation of data for the work; drafting the work or revising it critically for important intellectual content; final approval of the version to be published
GSC	Substantial contributions to the conception or design of the work; or the acquisition, analysis, or interpretation of data for the work; drafting the work or revising it critically for important intellectual content; final approval of the version to be published
NSM	Substantial contributions to the conception or design of the work; or the acquisition, analysis, or interpretation of data for the work; drafting the work or revising it critically for important intellectual content; final approval of the version to be published
ELSJ	Substantial contributions to the conception or design of the work; or the acquisition, analysis, or interpretation of data for the work; drafting the work or revising it critically for important intellectual content; final approval of the version to be published
CCVL	Substantial contributions to the conception or design of the work; or the acquisition, analysis, or interpretation of data for the work; drafting the work or revising it critically for important intellectual content; final approval of the version to be published
SMVS	Substantial contributions to the conception or design of the work; or the acquisition, analysis, or interpretation of data for the work; drafting the work or revising it critically for important intellectual content; final approval of the version to be published
